# Physicochemical gradients shape zooplankton and bivalve communities along the Sô River–Lake Nokoué continuum in Benin: implications for environmental health monitoring

**DOI:** 10.1007/s10661-026-16151-x

**Published:** 2026-11-13

**Authors:** Houéyi B. P. Capo-Chichi, Olaègbè Victor Okpeitcha, S. Arnaud S. Soha, Toussaint Mitchodigni, Mardochée Achoh, Zacharie Sohou, Victorien T. Dougnon, Anders Dalsgaard, Georges Degbe, Yaovi M. Gildas Hounmanou

**Affiliations:** 1 Laboratory of Hydrobiology and Aquaculture, Faculty of Agronomic Sciences, University of Abomey-Calavi, Abomey-Calavi, Benin; 2 Benin Institute of Fisheries and Oceanographic Research, Cotonou, Benin; 3 International Chair in Mathematical, Physics and Applications, University of Abomey-Calavi, Abomey-Calavi, Benin; 4 Research Unit in Applied Microbiology and Pharmacology of Natural Substances, Research Laboratory in Applied Biology, Polytechnic School of Abomey-Calavi, University of Abomey-Calavi, Abomey-Calavi, Benin; 5 Research Unit in Aquaculture and Fisheries Management, School of Aquaculture, National University of Aquaculture, Porto-Novo, Benin; 6 Faculty of Health and Medical Sciences, University of Copenhagen, Copenhagen, Denmark; 7 Department of Veterinary and Animal Sciences, Faculty of Health and Medical Sciences, Section of Bacteria and Viruses, University of Copenhagen, Copenhagen, Denmark

**Keywords:** Environmental gradients, Aquatic ecosystems, Climate change, Lake Nokoué, Sô River

## Abstract

Tropical river–lagoon systems are dynamic environments in which physicochemical variability can influence aquatic community structure. This study characterized the composition, taxonomic richness, occurrence, and spatiotemporal variation of zooplankton and bivalve communities along the Sô River–Lake Nokoué continuum in southern Benin. Monthly biological sampling was conducted at 11 stations from August 2024 to March 2025, including four stations in the Sô River (S1–S4) and seven in Lake Nokoué (S5–S11). A total of 24 zooplankton families and nine bivalve families were recorded. Eurotatoria dominated the zooplankton community, accounting for 56% of total relative density and exhibiting the highest taxonomic richness. Bivalves showed lower family-level richness but were recorded across the study area. Principal component analysis revealed a marked physicochemical gradient associated primarily with salinity, electrical conductivity, and total dissolved solids, distinguishing riverine from lagoonal conditions. Redundancy analysis identified associations between zooplankton community composition and dissolved oxygen and salinity, whereas bivalve community composition was associated with pH and temperature. These results highlight the environmental heterogeneity of the Sô River–Lake Nokoué continuum and provide an ecological baseline for future aquatic monitoring. Because pathogens, human exposure, and disease outcomes were not assessed, the findings do not establish a direct relationship between the observed community patterns and waterborne disease risk.

## Introduction

Continental tropical aquatic ecosystems play a central role in biodiversity conservation, food security, water provision, and the livelihoods of riparian populations (Chaigneau et al., 2023; Davies-Vollum et al., 2024). Beyond their ecological functions, these ecosystems also represent critical interfaces between environmental change and public health. In many low- and middle-income countries, degraded aquatic environments can contribute to the emergence and transmission of climate-sensitive waterborne diseases such as cholera, schistosomiasis, and other diarrheal infections (Colwell, 1996; Lipp et al., 2002; World Health Organization, 2023). Changes in water quality, hydrological regimes, and aquatic biodiversity can directly influence pathogen persistence, vector ecology, and human exposure risks within densely populated socioecological systems (Bekele et al., 2025; Vezzulli et al., 2016).

Zooplankton and benthic bivalves are increasingly recognized as ecological indicators and may also provide information relevant to environmental health and disease risk assessment (Jeppesen et al., 2011). Zooplankton play an essential role in aquatic food webs by transferring energy from primary producers to higher trophic levels, and benthic bivalves are used in filtration, organic matter recycling, and sediment digestion (Gosling, 2015). Other studies have also highlighted the important role of planktonic organisms in pathogen ecology, particularly through their interactions with *Vibrio cholerae* and other pathogenic bacteria in aquatic environments (de Magny et al., 2011; Erken et al., 2015; Perera et al., 2022). Copepods may serve as reservoirs or carriers of *Vibrio cholerae*, the cause of cholera in aquatic systems (Colwell, 1996; Huq et al., 1983). Similarly, bivalves may accumulate microorganisms and contaminants through filtration and therefore may represent microbial and environmental contamination in aquatic communities (Gosling, 2015).

In West Africa, lagoon and fluvio-lagoon environments are increasingly exposed to anthropogenic and climatic pressures for development and urbanization, nutrient enrichment, untreated wastewater discharge, flooding, salinity intrusion, and hydrological instability (Davies-Vollum et al., 2024; Djihouessi & Aina, 2018). Climate change exacerbates these disturbance factors with increasing temperatures, irregular rainfall, extreme flooding events, and extended stagnation periods that might contribute to pathogen spread and disease outbreaks (Bekele et al., 2025; Vezzulli et al., 2016). This environmental deterioration can harm the ecosystem and human health. Lake Nokoué is the largest lagoon in southern Benin and is one of the most vulnerable aquatic socioecological systems in the area. The lake undergoes strong seasonal hydrological changes with water inflow from the Sô River and marine intrusion into the Cotonou Channel (Sintondji et al., 2022). These processes influence salinity, turbidity, oxygenation, nutrient availability, and biological productivity (Chaigneau et al., 2023).

Previous ecological studies in Lake Nokoué have documented patterns in individual biological compartments. Zooplankton seasonal dynamics and abundance have been characterized in relation to salinity gradients and tidal exchanges (Adandédjan et al., 2017; Akodogbo et al., 2024; Chaigneau et al., 2023). Local studies have also highlighted the role of benthic macroinvertebrates in the lagoonal food web and have examined their spatial distribution in relation to organic pollution and ecological indices (Adandédjan, 2012; Capo-Chichi et al., 2022; Gnohossou, 2006; Gnohossou et al., 2009). However, previous studies have generally examined planktonic and benthic components separately, limiting an integrated assessment of how environmental gradients simultaneously structure multiple aquatic communities. In addition, much of the local literature has focused on pollution patterns or individual ecological metrics rather than linking community responses to environmental health along a river–lagoon continuum. A synchronized, multitaxon assessment of the Sô River–Lake Nokoué continuum can therefore provide complementary information on zooplankton and bivalve community structure and its relevance to environmental health monitoring.

This study addresses this gap by examining whether hydroclimatic and mineralization gradients act as environmental filters shaping zooplankton and bivalve communities along the Sô River–Lake Nokoué continuum. Specifically, we examine how changes in salinity, conductivity, temperature, and related physicochemical variables are associated with community composition, including the distribution of zooplankton and filter-feeding bivalves. Because this study did not directly measure pathogens, its public health relevance is considered from an ecological monitoring perspective rather than as direct evidence of pathogen occurrence or disease transmission. Although this study focuses on a Beninese river–lagoon continuum, the ecological processes examined may be relevant to other tropical transitional aquatic systems experiencing climate variability, high human pressure, and sanitation challenges. Understanding how environmental gradients structure aquatic communities can contribute to the development of integrated environmental monitoring approaches for climate-sensitive waterborne disease risk assessment.

Accordingly, this study aimed to characterize the ecological structure of zooplankton and bivalve communities along the Sô River–Lake Nokoué continuum and to assess their relevance for environmental health monitoring. Specifically, we sought to

(i) characterize the taxonomic composition, richness, occurrence, and spatiotemporal variation in the abundance of zooplankton and bivalve assemblages along the continuum;
(ii) characterize the hydroclimatic and physicochemical gradients using principal component analysis (PCA) and identify the main environmental gradients distinguishing riverine and lagoonal stations; and
(iii) assess the relationships between hydroclimatic variability, physicochemical conditions, and aquatic community structure using redundancy analysis (RDA), thereby evaluating the potential utility of these communities for environmental monitoring.

## Materials and methods

### Study area

The Nokoué–Sô River complex forms a major interconnected hydrological system in southern Benin that is crucial for fishing, aquaculture, and ecological balance. Lake Nokoué, a large lagoon with extensive rainfall and saltwater intrusion, is seasonal in nature (Sintondji et al., 2022). The Sô River is a major northern tributary of the lake, and the freshwater supply of the lake depends on the river in the flood phase and influences the salinity and water quality (Sintondji et al., 2022). The Sô River is rich in nutrients which help to feed the biological productivity of the lake in high biological productivity which is supported by the nutrients that are transferred from the Sô River. Biological communities, including phytoplankton, zooplankton, and macroinvertebrates, exhibit a considerable spatiotemporal variability with respect to environmental conditions. Lake Nokoué is also a key site of fishery production and is extensively exploited by local people with the use of the acadja (Zandagba et al., 2016). However, these environments are also increasingly subject to anthropogenic factors such as domestic wastewater, agricultural activities, and urbanization. This is producing water quality degradation leading to eutrophication and decreasing dissolved oxygen (Zandagba et al., 2016).

### Sampling stations

**Fig. 1:**
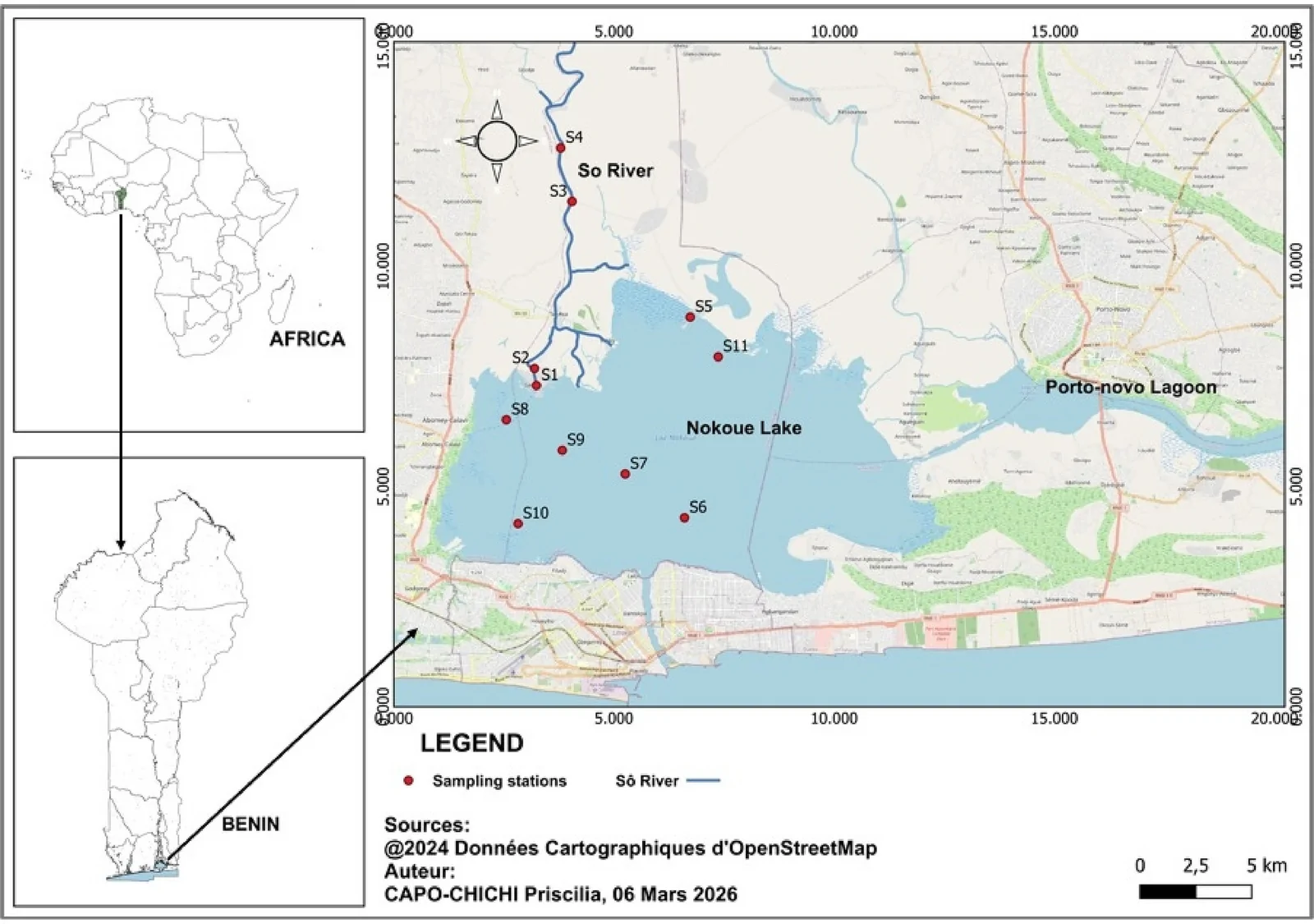
Location of the 11 sampling stations along the Sô River–Lake Nokoué continuum in southern Benin

Physicochemical and biological data were collected monthly at 11 strategically selected stations (Fig. 1): four stations along the freshwater gradient of the Sô River (S1–S4) and seven stations across the brackish lagoonal expanse of Lake Nokoué (S5–S11). Stations were selected based on accessibility, habitat heterogeneity, and proximity to lacustrine settlements. GPS coordinates were recorded at the center of each station during every field campaign using a Garmin GPSMAP 64 s unit to ensure spatial consistency. Table 1 presents the geographic coordinates of all sampling stations. All field observations and biological collections were conducted within a standardized sampling window from 06:00 to 09:30 to minimize potential effects of diel thermal variation and vertical migration.

**Table 1:** Geographic coordinates of all sampling stations

Stations	Geographic coordinates	
	*X*	*Y*
S1	06°28′04.152″N	002°23′29.148″E
S2	06°28′29.064″N	002°23′26.376″E
S3	06°32′33.000″N	002°24′20.952″E
S4	06°33′51.048″N	002°24′04.284″E
S5	06°29′44.196″N	002°27′13.608″E
S6	06°24′51.228″N	002°27′05.724″E
S7	06°25′55.020″N	002°25′39.036″E
S8	06°27′13.932″N	002°22′45.444″E
S9	06°26′29.472″N	002°24′07.164″E
S10	06°24′42.192″N	002°23′02.544″E
S11	06°28′46.272″N	002°27′54.504″E

Sampling was synchronized with seasonal meteorological and hydroclimatic conditions. Sampling from August to November covered the peak rainy season, intense continental runoff, and subsequent flooding events, whereas December to March represented the dry season, characterized by lower river discharge and stronger marine influence (Chaigneau et al., 2023). To account for short-term hydrodynamic variability, sampling events were standardized across all stations during high-tide or slack-water conditions to minimize localized transport effects and ensure comparable water-column measurements (Akodogbo et al., 2024).

### Measurement of physicochemical parameters

Physicochemical parameters were measured monthly from August 2024 to January 2025 in situ using an Aquaread multiparameter water quality probe (AP-800D). The parameters measured were water temperature, salinity, turbidity, conductivity, total dissolved solids (TDS), dissolved oxygen, oxygen at saturation, depth, and pH.

### Biological data sampling

Biological data were collected monthly from August 2024 to March 2025. Zooplankton were collected using a plankton net with a 40-cm aperture and a 50-µm mesh size towed horizontally over a distance of at least 15 m. After filtration, samples were concentrated in labeled containers. In the laboratory, quantitative and qualitative analyses were conducted with a KERN OBE 134T241 microscope on 1 mL subsamples that were well homogenized, with three replicates (total volume of 3 mL per sample).

Zooplankton were identified and counted using standard identification keys (Durand & Lévêque, 1981). The zooplankton density (*D*) was calculated to determine the number of individuals per liter (ind./L), following Monney et al., 2016 using the formula *D* = (*n* × *V*_2_)/(*V*_1_ × *V*_3_), where *n* is the number of individuals of a given taxon (or total zooplankton) counted in the examined subsample, *V*_1_ is the volume of the subsample (aliquot) examined under the microscope (i.e., in mL), *V*_2_ is the final volume of the concentrated sample after filtration (i.e., in mL), and *V*_3_ is the initial volume of water filtered in the field (i.e., the total volume of raw water passed through the net, typically in L). The volume of water filtered at each station was recorded using a calibrated mechanical flowmeter and was used to estimate zooplankton density.

Bivalves were sampled using an Eckman grab (Adandédjan, 2012; Capo-Chichi et al., 2022). At each station, the grab was used five times, and the collected material was washed with site water and sieved through a 1-mm mesh. The samples were preserved in 10% formalin in labeled plastic containers. In the laboratory, samples were rinsed with tap water through a series of 1 mm and 500 µm mesh sieves to remove fine sediments and debris (gravel, plants, leaves). Organisms were sorted using forceps under a binocular magnifying glass. Bivalves were separated based on morphological characteristics and grouped according to class, order, and family. The identification and counting of the samples were carried out with a Nikon binocular microscope and standard identification keys (Durand & Lévêque, 1981; Tachet et al., 2006; Moisan, 2010). Taxonomic identification was done to family level for bivalves and zooplankton.

### Data analysis

The composition of zooplankton and bivalve communities was then determined using taxonomic richness, occurrence frequency, relative density (zooplankton), and abundance (bivalves). The occurrence of the zooplankton and bivalve families for each sampling station was evaluated using data from August 2024 to March 2025. Their percentage (*F*) was taken to describe habitat preferences and calculated as (Dajoz, 2000) *F* = Fi × 100/Ft where Fi is the number of samples with family i and Ft is the total number of samples.

#### Diversity indices

To complement the analysis of taxonomic richness, abundance, and occurrence frequency, Shannon diversity (*H*′) and Pielou’s evenness (*J*′) were calculated separately for zooplankton and bivalve assemblages at the family level. For each station and sampling month, Shannon diversity was calculated as *H*′ =  − Σ(*pᵢ* ln *pᵢ*), where *pᵢ* = *nᵢ*/*N*, *nᵢ* is the abundance of taxon *i*, and *N* is the total abundance of all taxa in the sample. The Shannon index was calculated using the natural logarithm (ln) and is therefore expressed in nats. Pielou’s evenness was calculated as *J*′ = *H*′/ln(*S*), where *S* is the number of taxa (families) observed in the sample. Pielou’s evenness ranges from 0 to 1, with higher values indicating a more even distribution of abundances among the taxa present. Diversity indices were calculated separately for each sampling station and month.

Principal component analysis (PCA) was performed using the FactoMineR package in R (RStudio environment). Principal component analysis (PCA) was performed using physicochemical parameter values to establish the abiotic typology of the different sampling stations in the study area. This analysis identified similarities among stations within the lagoon complex based on environmental parameters. The input matrix consisted of environmental data comprising 9 parameters × 6 months × 11 stations.

Redundancy analysis (RDA) was used to investigate the relationships between environmental conditions and the composition of zooplankton and bivalve communities. Before conducting the RDA, detrended correspondence analysis (DCA) was performed separately on each biological dataset to assess the length of the compositional gradient. The first-axis gradient lengths were 1.553 standard deviation (SD) units for zooplankton and 1.047 SD units for bivalves, supporting the use of a linear ordination approach.

Biological community matrices were constructed at the station–month level. Taxa recorded at fewer than five sampling stations were excluded to limit the influence of rare taxa. The zooplankton and bivalve community matrices were then Hellinger-transformed before analysis to reduce the influence of highly abundant taxa and make the data suitable for linear constrained ordination. The initial environmental predictor set comprised temperature, salinity, turbidity, electrical conductivity, total dissolved solids (TDS), dissolved oxygen, oxygen saturation, pH, and depth. Because electrical conductivity, TDS, and salinity represent related aspects of water mineralization and dissolved oxygen and oxygen saturation represent related aspects of oxygen conditions, alternative predictor combinations were evaluated to assess the stability of variable selection and model performance. Variance inflation factors (VIFs) were examined in the selected models to assess multicollinearity. Forward selection was used to identify parsimonious sets of environmental predictors. Six alternative candidate-predictor combinations were compared for each biological community, replacing the mineralization proxy (salinity, conductivity, or TDS) and the oxygen-related predictor (dissolved oxygen or oxygen saturation). Model selection considered adjusted *R*^2^, predictor-level significance, parsimony, and consistency across alternative combinations rather than relying solely on the highest explained variance. The final reference models were evaluated using permutation tests with 999 permutations restricted within sampling stations to account for repeated sampling at the same stations. The significance of each model as a whole, its constrained axes, and the individual environmental predictors was assessed.

All statistical analyses were performed using R software (version 4.5.1) in the RStudio environment, and statistical significance was established at *P* < 0.05.

## Results and discussion

### Composition of zooplankton and bivalve communities

A total of 24 zooplankton families were recorded across the Sô River–Lake Nokoué complex (Table 2). These families belonged to five orders (Anomopoda, Calanoida, Cyclopoida, Flosculariaceae, and Ploima) and three classes (Branchiopoda, Copepoda, and Eurotatoria). Most families were classified as incidental (70.8%), whereas 29.2% were accessory. Accessory families included Daphniidae, Moinidae, Cyclopidae, Filiniidae, Brachionidae, Lecanidae, and Synchaetidae.

**Table 2:** Occurrence of zooplankton families recorded at sampling stations in the Sô River (S1–S4) and Lake Nokoué (S5–S11)

Class	Order	Family	S1	S2	S3	S4	S5	S6	S7	S8	S9	S10	S11	%*F*
Branchiopoda	Anomopoda	Bosminidae	*	*		*	*	*	*	*	*	*	*	21.5
		Daphniidae	*	*	*	*	*	*	*	*	*	*	*	47.7
		Macrothricidae	*	*		*	*		*		*			10.2
		Moinidae	*	*	*	*	*	*		*	*	*	*	25.0
Copepoda	Calanoida	Acartiidae	*	*		*			*	*	*		*	13.6
		Calanidae			*			*	*	*	*	*	*	11.3
		Diaptomidae	*	*	*	*				*				6.8
		Pseudodiaptomidae	*	*		*			*		*		*	12.5
	Cyclopoida	Cyclopidae	*	*	*	*	*	*	*	*	*		*	30.6
		Oithonidae	*	*		*			*				*	7.95
Eurotatoria	Flosculariaceae	Conochilidae					*	*	*	*	*	*	*	10.2
		Filiniidae	*	*	*	*	*	*	*	*	*	*	*	40.9
		Hexarthridae	*		*	*	*	*	*	*		*	*	18.1
		Testudinellidae	*		*	*	*	*	*	*		*	*	20.4
	Ploima	Asplanchnidae	*	*		*	*	*	*	*	*	*	*	23.8
		Brachionidae	*	*	*	*	*	*	*	*	*	*	*	46.5
		Epiphanidae			*		*	*	*		*	*		9.09
		Euchlanidae	*	*	*	*	*	*	*	*	*	*	*	18.1
		Lecanidae	*		*	*	*	*	*	*	*	*	*	47.7
		Lepadellidae	*		*	*	*	*	*	*		*	*	17.0
		Mytilinidae	*		*	*	*	*	*	*	*	*	*	20.4
		Notommatidae					*	*	*			*		6.8
		Synchaetidae	*	*	*	*	*	*	*	*	*	*	*	36.3
		Trichocercidae	*		*	*	*	*		*	*	*	*	22.7

* indicates presence of a family at a station; *F* (%) indicates frequency of occurrence across all sampling events

Bivalve assemblages were less diverse, with nine families distributed across eight orders (Arcida, Myoida, Myida, Mytilida, Ostreida, Cardiida, Unionida, and Veneroida) (Table 3). In contrast to zooplankton, bivalves included one constant family, Sphaeriidae (11.1%), while 44.4% of families were accessory (Corbulidae, Mytilidae, Ostreidae, and Unionidae) and 44.4% were incidental.

**Table 3:** Occurrence of bivalve families recorded at the 11 sampling stations along the Sô River and Lake Nokoué

Class	Order	Family	S1	S2	S3	S4	S5	S6	S7	S8	S9	S10	S11	*F* (%)
Bivalvia	Arcida	Arcidae			*	*	*	*	*	*	*	*	*	21.6
Bivalvia	Myoida	Corbulidae	*	*	*	*	*	*	*	*	*	*	*	40.9
Bivalvia	Myida	Dreissenidae	*	*	*	*	*	*	*	*	*	*	*	23.9
Bivalvia	Mytilida	Mytilidae	*	*	*	*	*	*	*	*	*	*	*	38.6
Bivalvia	Ostreida	Ostreidae	*	*	*	*	*	*	*	*	*	*	*	42.0
Bivalvia	Cardiida	Solecurtidae		*			*	*		*	*	*	*	12.5
Bivalvia	Cardiida	Tellinidae	*	*	*		*		*	*	*	*	*	22.7
Bivalvia	Unionida	Unionidae	*	*	*	*	*	*	*	*	*	*	*	46.6
Bivalvia	Veneroida	Sphaeriidae	*	*	*	*	*	*	*	*	*	*	*	65.9

* indicates presence of a family at a given station; *F* (%) indicates frequency of occurrence

These patterns indicate marked differences in the ecological structure of planktonic and benthic communities. The relatively high richness of zooplankton, dominated by rotifers, cladocerans, and copepods, is consistent with patterns reported for tropical aquatic systems, where these groups typically constitute the major components of the plankton community (Segers, 2008). The prominence of Ploima and Cyclopoida further suggests productive conditions, potentially associated with nutrient enrichment and high organic matter availability. The high proportion of incidental zooplankton families indicates substantial spatial and/or temporal variability in environmental conditions. Such a pattern is typical of ecosystems subject to hydrological fluctuations and anthropogenic disturbance, where many taxa occur only under temporarily favorable conditions (Allan & Castillo, 2007; Jeppesen et al., 2011). By contrast, the recurring presence of Daphniidae, Cyclopidae, Brachionidae, and other accessory families suggests that these taxa are more tolerant of environmental heterogeneity and are able to persist under a wider range of conditions. The lower taxonomic richness of bivalves is expected, given that benthic communities are generally less diverse than zooplankton but often more stable over time. The occurrence of Sphaeriidae as the only constant family suggests broad tolerance to local habitat conditions, including variation in oxygenation, turbidity, and substrate characteristics. This is consistent with previous observations that Sphaeriidae can colonize a wide range of habitats, including disturbed or organically enriched environments (Adandédjan, 2012). The equal proportions of accessory and incidental bivalve families also indicate environmental heterogeneity along the continuum. The coexistence of freshwater-associated taxa such as Unionidae and Sphaeriidae with more salinity-tolerant families such as Mytilidae and Ostreidae points to a marked ecological transition between riverine and lagoonal conditions. Such distributions are characteristic of transitional aquatic systems, where biodiversity reflects the overlap of taxa with contrasting ecological requirements (Attrill & Rundle, 2002; Gosling, 2015). Overall, the composition of both zooplankton and bivalve communities supports the view that the study area is a dynamic and environmentally variable system influenced by both continental and lagoonal processes.

### Taxonomic richness and relative density of zooplankton orders

**Fig. 2:**
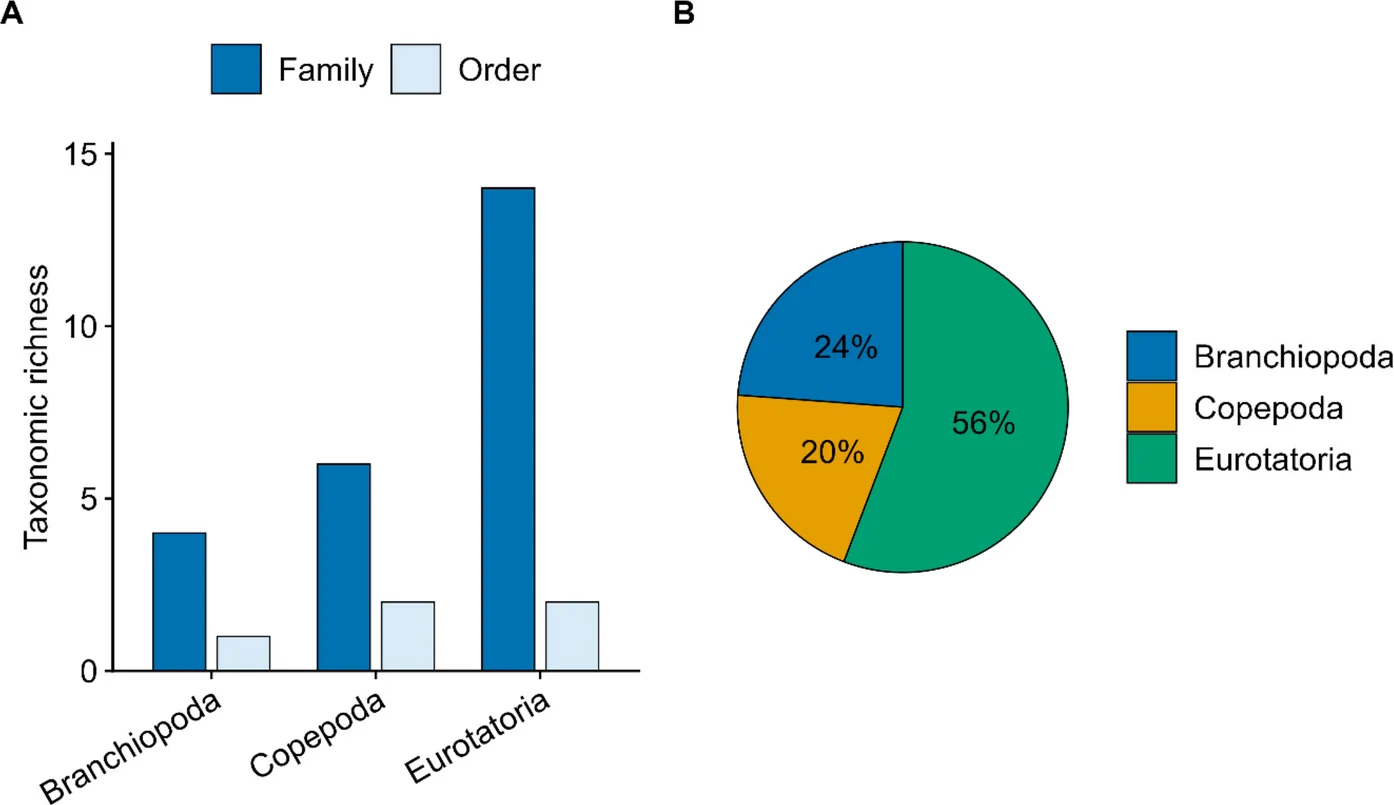
Taxonomic richness at family and order levels and relative density of the major zooplankton groups along the Sô River–Lake Nokoué continuum. **A** Taxonomic richness at family and order levels. **B** Relative density of Branchiopoda, Copepoda, and Eurotatoria

The taxonomic composition of the zooplankton community showed clear differences among the three groups identified (Fig. 2A). Eurotatoria displayed the highest taxonomic richness (2 orders and 14 families) and the greatest relative abundance, accounting for 56% of the total density. Copepoda ranked second in richness (2 orders and 6 families) but represented only 20% of the total density, while Branchiopoda exhibited the lowest richness (1 order and 4 families) and contributed 24% of the total density (Fig. 2B). These results highlight the dominance of Eurotatoria in both diversity and abundance within the studied zooplankton community.

The results of Lake Nokoué and the Sô River complex showed Eurotatoria as dominant in both taxonomic richness (2 orders and 14 families) and relative density (56%). This is common in tropical aquatic ecosystems due to the short life cycle, high reproductive capacities, and fast resource use of rotifers (Chaigneau et al., 2023; Stamou et al., 2023). Their high richness could also be related to the nutrient-rich environment of the Nokoué–Sô system, which allows rotifers that are usually associated with eutrophic and mesotrophic environments (Adandédjan et al., 2017; Chaigneau et al., 2023). Copepods (2 orders and 6 families) have a high taxonomic richness but a low relative density (20%), possibly because they have a greater sensitivity to environmental factors such as salinity, temperature, and food availability (Akodogbo et al., 2024; Chaigneau et al., 2023). Branchiopods (1 order and 4 families) have the lowest taxonomic richness but contribute to total density (24%) so it is expected that some cladocerans are particularly well adapted to local conditions and are able to take advantage of the available trophic resources (Gavrilko et al., 2024; Stamou et al., 2023). The zooplankton community structure is indicative of a productive ecosystem with abundant trophic resources, where rotifer dominance indicates nutrient-rich environments, as found in West African aquatic systems (Adandédjan et al., 2017; Chaigneau et al., 2023; Dumont, 1994).

### Monthly variation in relative density and richness of zooplankton

**Fig. 3:**
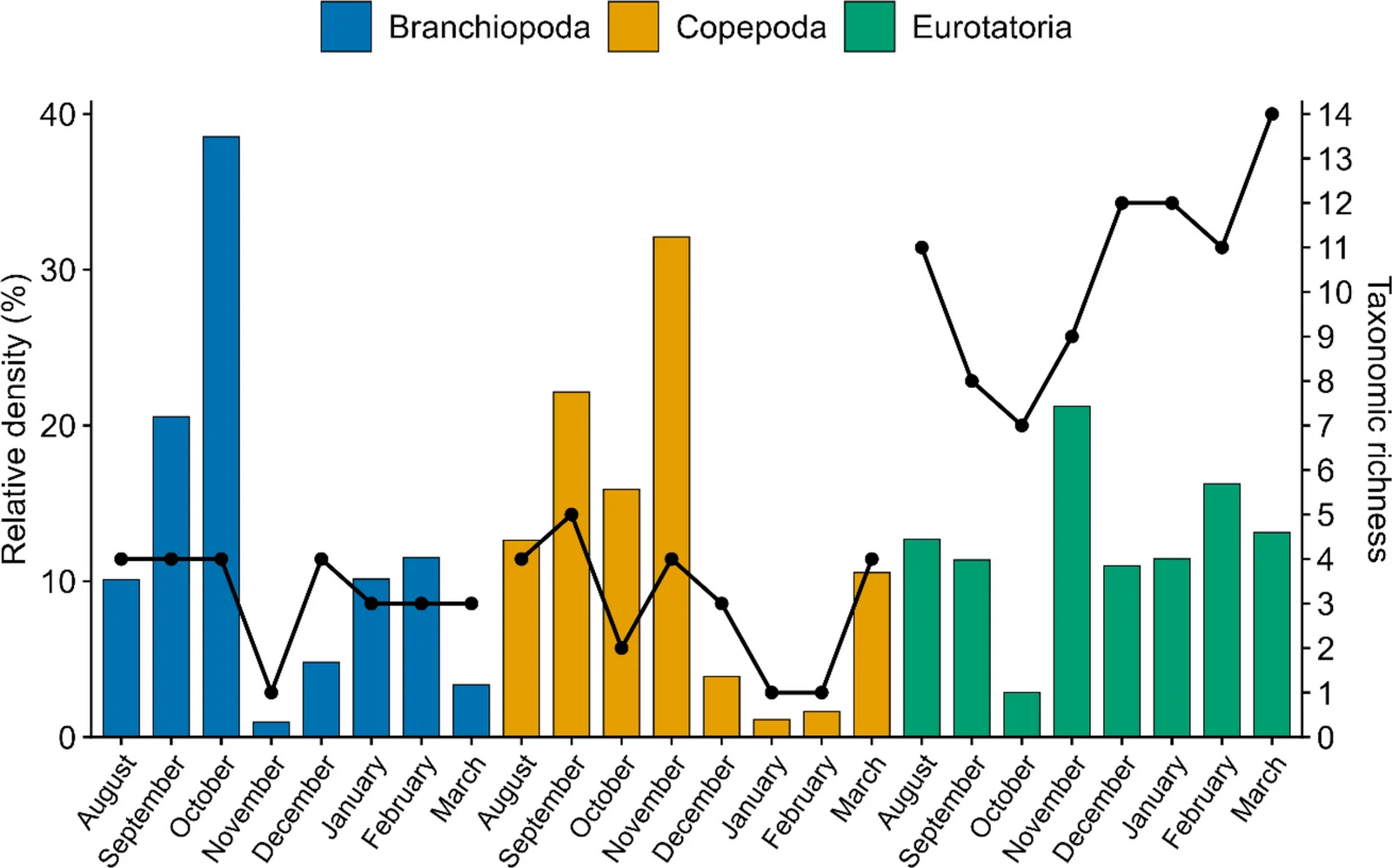
Monthly variation in relative density and richness of zooplankton

The monthly variation in zooplankton communities from August to March showed distinct patterns among the three major groups (Fig. 3). Branchiopods reached their highest relative density in October while maintaining low and relatively stable taxonomic richness, suggesting a seasonal proliferation under favorable environmental conditions. Copepods were most abundant in September and November but experienced a marked decline in January and February, indicating sensitivity to seasonal changes despite limited variation in richness. In contrast, Eurotatoria displayed a progressive increase in both richness and relative density from January to March and remained abundant throughout the study period.

The monthly fluctuations in zooplankton richness and relative abundance from August to March are indicative of seasonal environmental changes on aquatic communities in Lake Nokoué and the Sô River (Cabral et al., 2020). Branchiopoda reached their highest relative density in October, likely due to increased phytoplankton production following nutrient inputs and runoff. There is still a high abundance in their taxonomic richness which suggests that only a few species are well adapted to local conditions. Copepoda are high in September and November followed by low abundance in January and February. As for Eurotatoria, their richness and density increased from January to March and remained abundant throughout the study period. Despite this, their dominance is due to their high ecological plasticity, short generation time, and response to favorable environmental conditions (Liu et al., 2023; Phan et al., 2021). The growth of rotifers toward the end of the study period may indicate more food and nutrient availability. As well-known bioindicators of trophic changes and eutrophication, their abundance is associated with high levels of organic matter and dissolved nutrients in aquatic ecosystems (Bhandarkar & Bansod, 2025).

### Spatial variation in relative density and richness of zooplankton

**Fig. 4:**
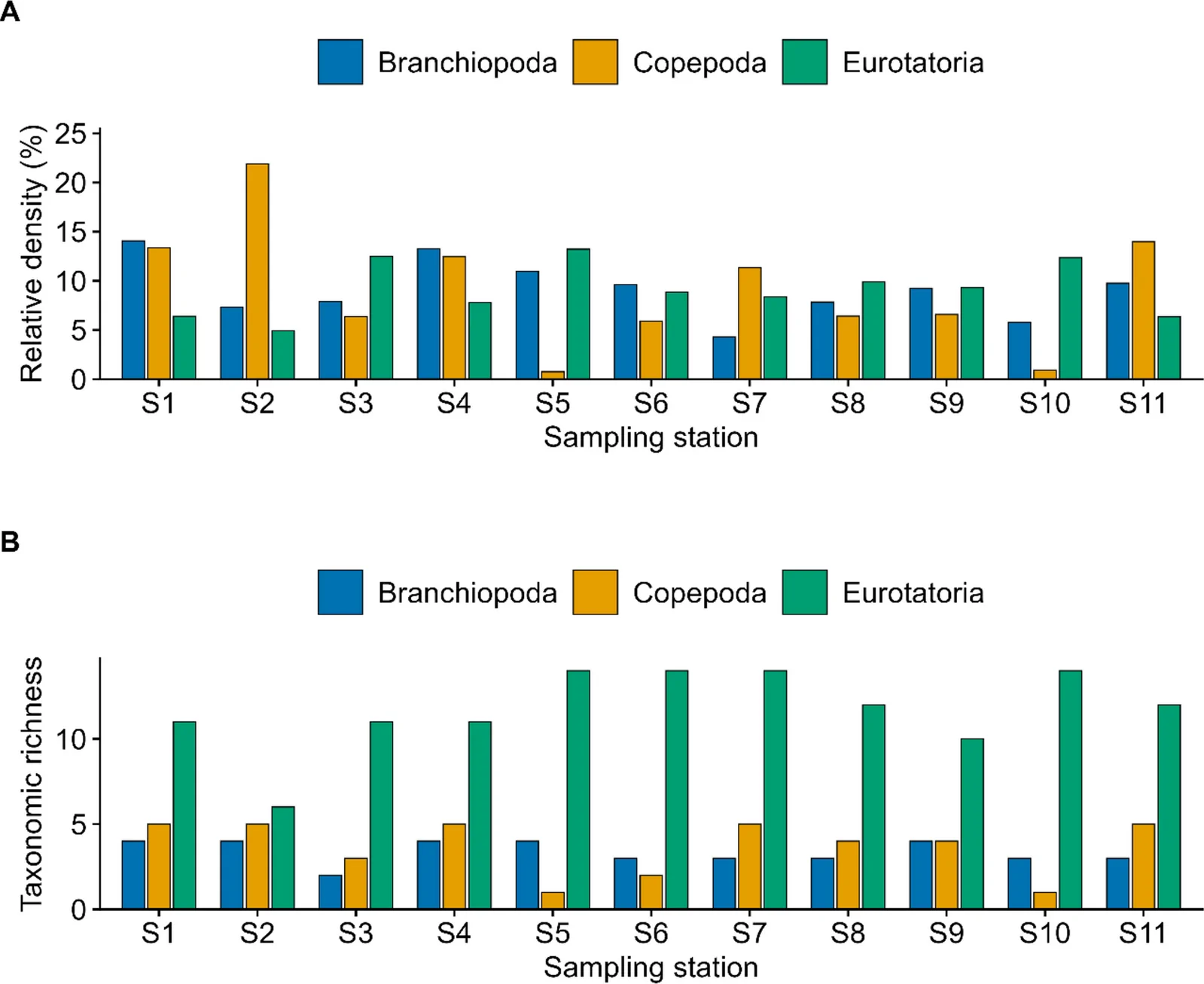
Spatial variation in the relative density and taxonomic richness of the zooplankton groups across sampling stations along the Sô River–Lake Nokoué continuum. **A** Relative density (%) of Branchiopoda, Copepoda, and Eurotatoria across sampling stations. **B** Taxonomic richness of the three groups across sampling stations

The distribution of zooplankton varied across the sampling stations in the Sô River and Lake Nokoué. Branchiopoda had relatively high density at some stations (Fig. 4A) and S1 and S4 but remained very stable and low in diversity in the entire study area (Fig. 4B). Copepoda were more diverse in distribution, and there was a clear abundance peak at S2 which indicates the influence of local environment. In contrast, Eurotatoria were extremely rich in taxonomic diversity and had relatively equal densities at most stations.

The spatial distribution of zooplankton communities observed in this study indicates that zooplankton communities are unevenly distributed between the Sô River and Lake Nokoué and that habitat characteristics and local environmental conditions (hydrodynamics, nutrients, and habitat connectivity) are in different directions among them (Cabral et al., 2020). Branchiopoda are found to be relatively high in density at stations S1 and S4 that would indicate favorable habitat conditions for cladoceran development, likely due to the abundance of food and reduced water turbulence. The low richness of Branchiopoda at stations S1 and S4 and the high local abundance indicate that few well-adapted taxa are also dominant (Ramos et al., 2022). Copepoda are heterogeneous and dense at station S2 and may be sensitive to local environmental factors such as water quality, hydrological conditions, and resource availability (Dehingia et al., 2026; Wagaw et al., 2023). Eurotatoria are a rich and relatively uniform group at most stations, indicating that they are able to colonize a wide range of habitats in a short time and possess widespread distribution and a high reproduction rate (Gavrilko et al., 2024; Umi et al., 2024). These differences may also reflect the contrasting ecological characteristics of riverine and lacustrine environments, where environmental filtering and habitat connectivity play an important role in structuring zooplankton communities (Aggio et al., 2022).

### Monthly and spatial variation in taxonomic richness and relative abundance of bivalves

**Fig. 5:**
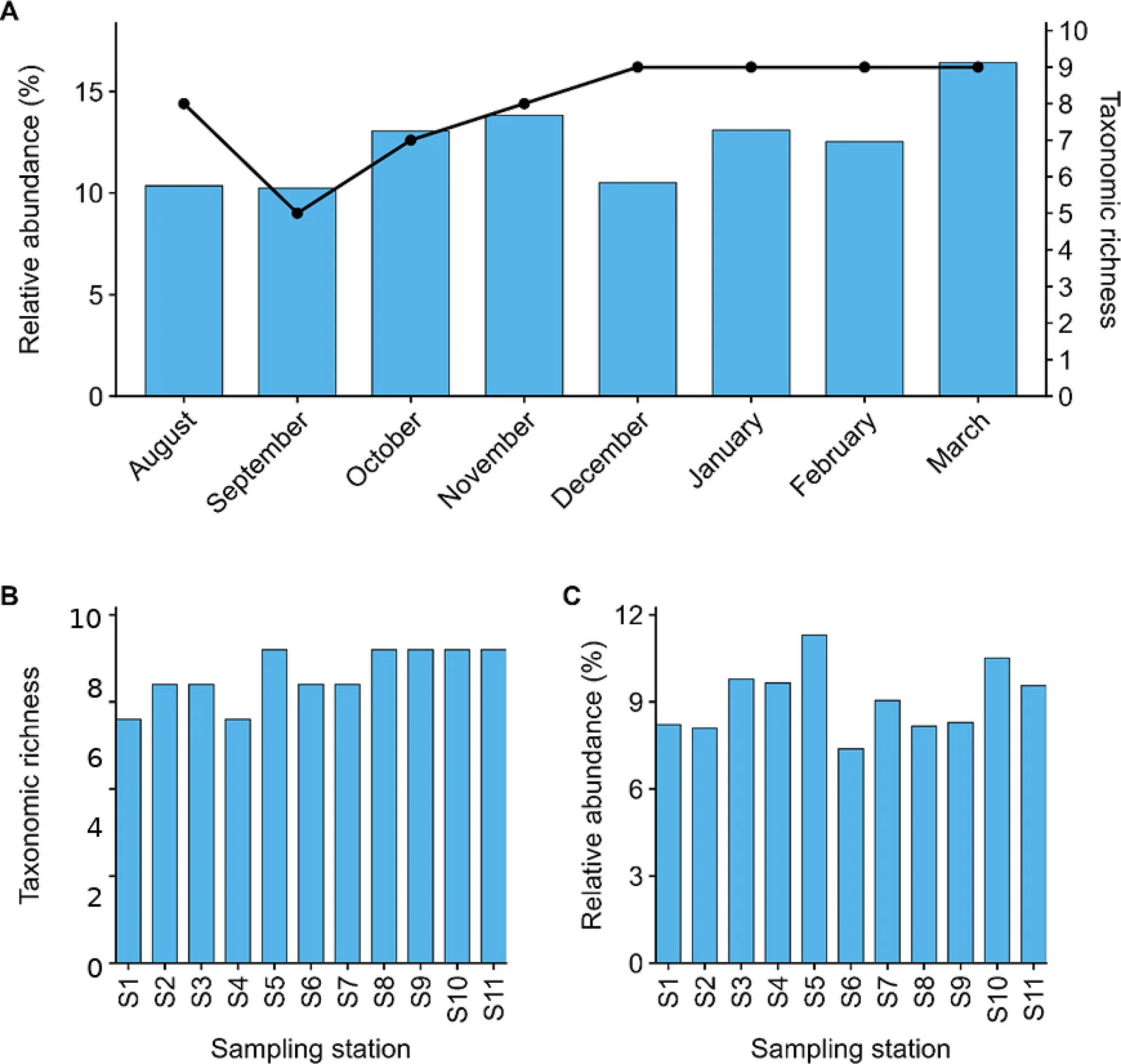
Monthly and spatial variation in bivalve taxonomic richness and relative abundance. **A** Monthly variation in taxonomic richness and relative abundance. **B** Spatial variation in taxonomic richness. **C** Spatial variation in relative abundance across sampling stations

The relative abundance of bivalves was observed to vary from 1 month to the next and at best in October, November, January, and March (Fig. 5A). Taxonomic richness remained relatively stable but increased significantly toward the end of our study (Fig. 5B). In this way, the seasonal environmental conditions became more favorable for the development of bivalves and increased abundance as a result. Bivalve abundance varied between the sampling stations, with the highest values at S5 and S10 and lowest values in S6 and S8 (Fig. 5C). Taxonomic richness also did not differ across stations, indicating that there was a wide range of taxa in the area. The observed differences in abundance can probably be explained by the local environment including habitat quality, substrate, and food availability.

The spatial variation of bivalve abundance and richness across months indicates that seasonal environmental factors are very much involved in the community dynamics. The increase in abundance during some months could be because of favorable conditions in the environment, better food supply, and increased primary productivity for bivalves (Sousa et al., 2014; Vaughn, 2018). The relatively stable richness observed in the study indicates that most taxa still existed throughout the year and seasonal changes only affected their population size. Similar patterns have been found in tropical aquatic ecosystems, where water level, nutrient and organic matter levels are controlled to regulate the abundance of benthic invertebrates without altering species composition (Bogan, 2008; Ge et al., 2025; Mohammed et al., 2020). The differences in bivalve abundance between sampling stations may be due to the local habitats and conditions. Bivalves are strongly influenced by substrate type, water depth, current velocity, and organic matter availability which determine the habitats for settlement and survival (Strayer, 2008; Vaughn, 2018). The relatively uniform richness across stations suggests that most taxa are spread across the Nokoué–Sô system and the environment is responsible for their abundance. Similar spatial patterns have been found in freshwater systems where habitat quality and availability of resources lead to differences in the number of bivalves whereas species richness is similar between sites (Lopes-Lima et al., 2017; Sousa et al., 2014).

### Diversity patterns of zooplankton and bivalve assemblages

#### Spatial and temporal variation in bivalve diversity

**Fig. 6:**
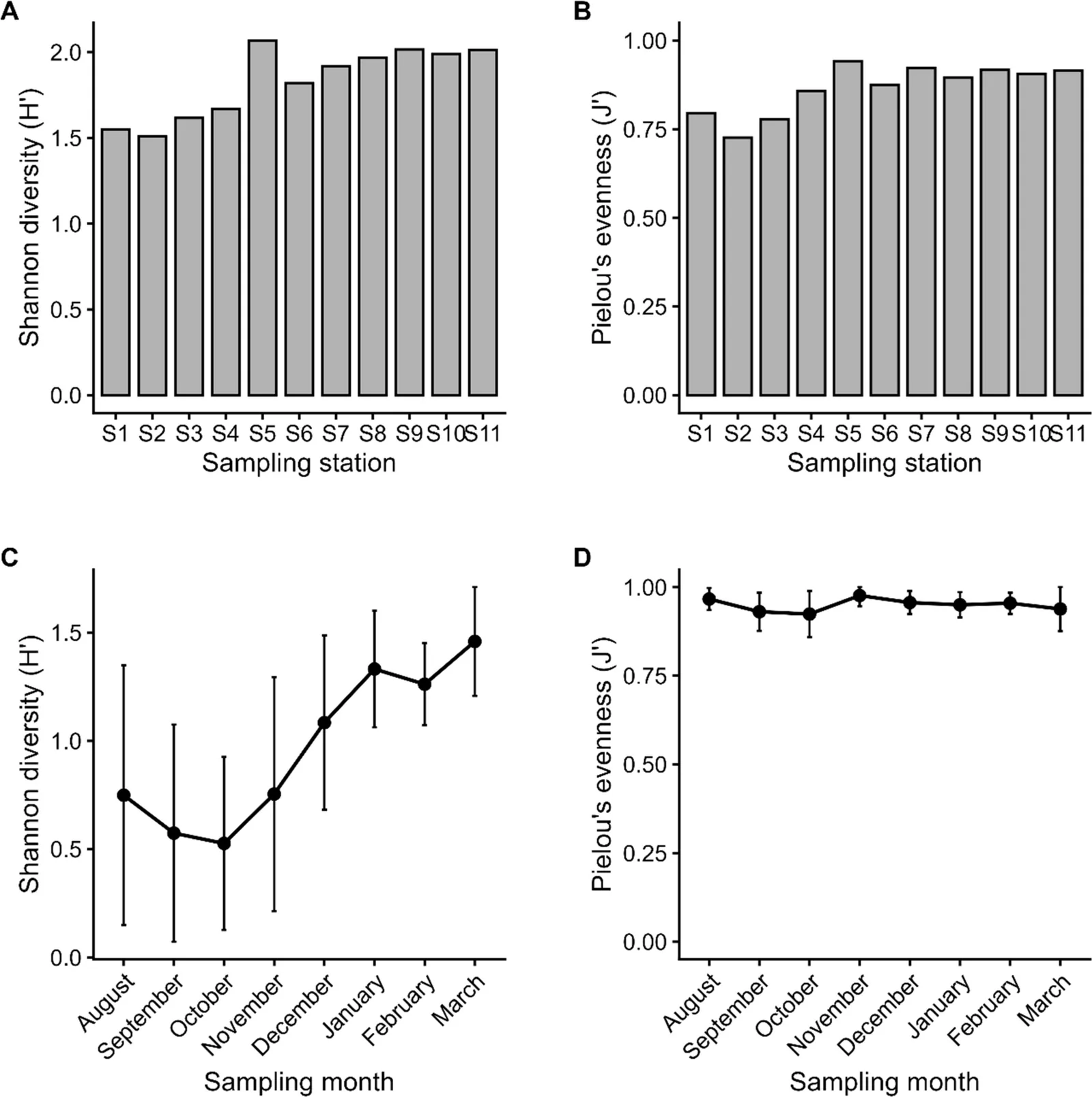
Spatial and temporal variation in bivalve diversity along the Sô River–Lake Nokoué continuum. **A** Spatial variation in Shannon diversity (*H*′) across sampling stations. **B** Spatial variation in Pielou’s evenness (*J*′). **C** Temporal variation in Shannon diversity (*H*′) from August 2024 to March 2025. **D** Temporal variation in Pielou’s evenness (*J*′). Spatial diversity indices were calculated from bivalve family-level abundances pooled across the sampling period, whereas temporal indices were calculated for each sampling month. Points and error bars in panels (**C**) and (**D**) represent monthly mean values ± standard deviation across sampling stations

Shannon diversity and Pielou’s evenness revealed distinct spatial and temporal patterns in the bivalve community (Fig. 6). Spatially, Shannon diversity ranged from 1.51 at S2 to 2.07 at S5 (Fig. 6A), indicating heterogeneity in bivalve diversity among sampling stations. Diversity was comparatively high at S5 and remained relatively elevated from S6 to S11, whereas S2 exhibited the lowest value. Pielou’s evenness ranged from 0.73 at S2 to 0.94 at S5 (Fig. 6B), with relatively high values at most stations, indicating generally even distributions of abundances among the taxa present.

Temporally, Shannon diversity showed pronounced variation throughout the sampling period (Fig. 6C). Mean *H*′ decreased from approximately 0.75 in August 2024 to a minimum of 0.53 in October 2024, followed by a progressive increase during the subsequent months, reaching approximately 1.46 in March 2025. A slight decline was observed in February 2025 before the increase in March. In contrast, Pielou’s evenness remained comparatively stable and high throughout the sampling period, ranging from approximately 0.92 in October 2024 to 0.98 in November 2024 (Fig. 6D). Overall, the stronger temporal variation in Shannon diversity than in Pielou’s evenness suggests that changes in bivalve diversity were not accompanied by major changes in the relative distribution of abundances among the taxa present.

#### Spatial and temporal variation in zooplankton diversity

**Fig. 7:**
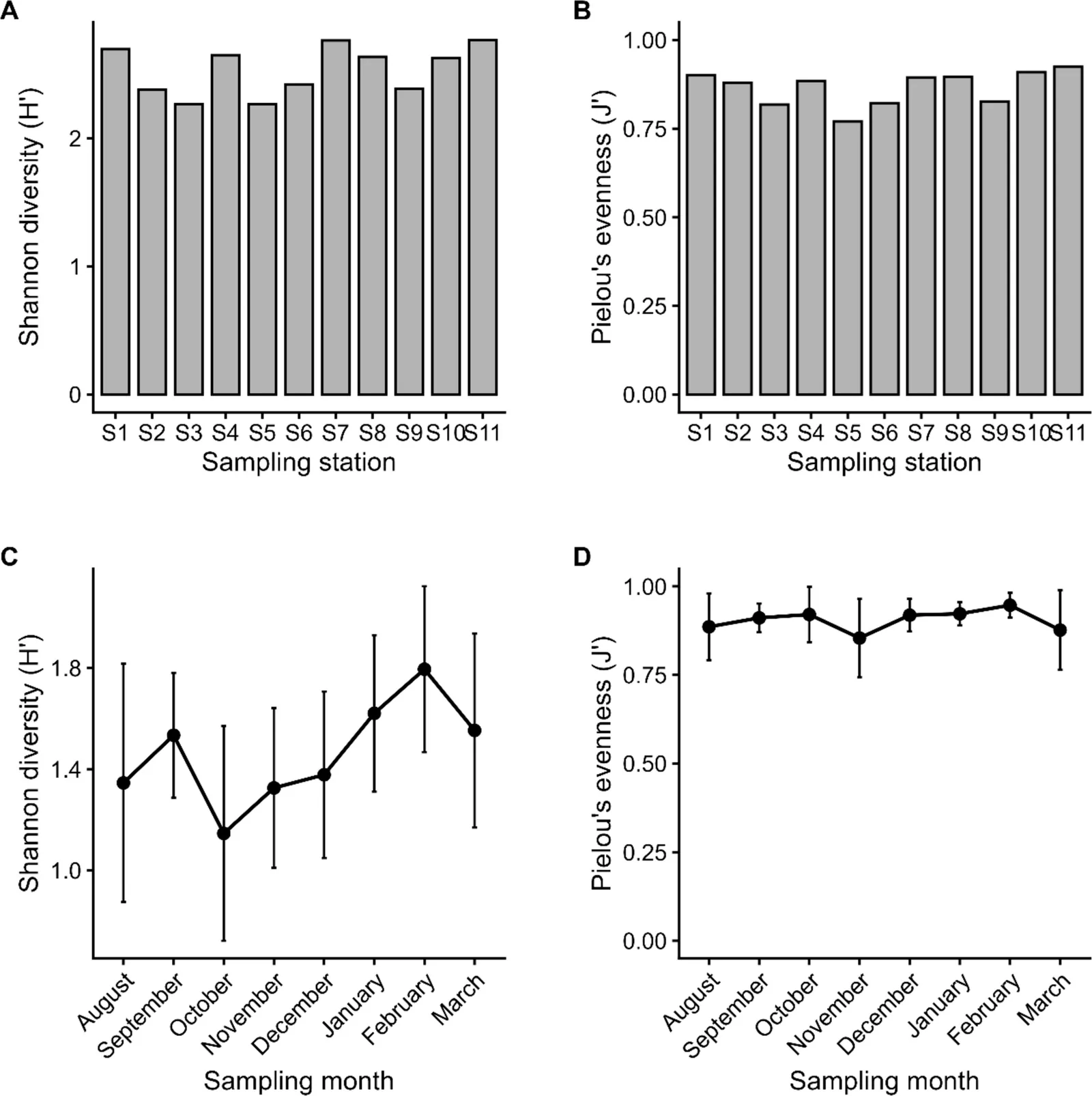
Spatial and temporal variation in zooplankton diversity along the Sô River–Lake Nokoué continuum. **A** Spatial variation in Shannon diversity (*H*′) across sampling stations. **B** Spatial variation in Pielou’s evenness (*J*′). **C** Temporal variation in Shannon diversity (*H*′) from August 2024 to March 2025. **D** Temporal variation in Pielou’s evenness (*J*′). Spatial diversity indices were calculated from zooplankton family-level abundances pooled across the sampling period, whereas temporal indices were calculated for each sampling month. Points and error bars in panels (**C**) and (**D**) represent monthly mean values ± standard deviation across sampling stations

Shannon diversity and Pielou’s evenness revealed distinct spatial and temporal patterns in the zooplankton community (Fig. 7). Spatially, Shannon diversity varied among sampling stations, ranging from approximately 2.3 at S5 to 2.8 at S11, with relatively high values also observed at S1, S4, S7, S8, and S10 (Fig. 7A). In contrast, the lowest diversity values were observed at S3 and S5. Pielou’s evenness ranged from approximately 0.78 at S5 to 0.93 at S11 (Fig. 7B). Most stations showed relatively high evenness, indicating a generally balanced distribution of abundances among the zooplankton taxa, although S5 and S9 exhibited comparatively lower evenness.

Temporally, Shannon diversity also varied throughout the sampling period. Mean *H*′ was approximately 1.35 in August 2024, increased to 1.55 in September, and subsequently declined to approximately 1.15 in October (Fig. 7C). Thus, zooplankton diversity showed a marked temporal fluctuation, with a minimum in October and a maximum in February.

Pielou’s evenness remained relatively high throughout the sampling period, ranging approximately from 0.85 in November 2024 to 0.94 in February 2025 (Fig. 7D). Evenness increased from August to October, followed by a decline in November and relatively high and stable values from December to February. A slight decrease was observed in March. Overall, the relatively limited temporal variation in Pielou’s evenness compared with Shannon diversity suggests that changes in zooplankton diversity were not associated with major shifts in the relative distribution of abundances among the taxa present.

### Principal component analysis of environmental variables

**Fig. 8:**
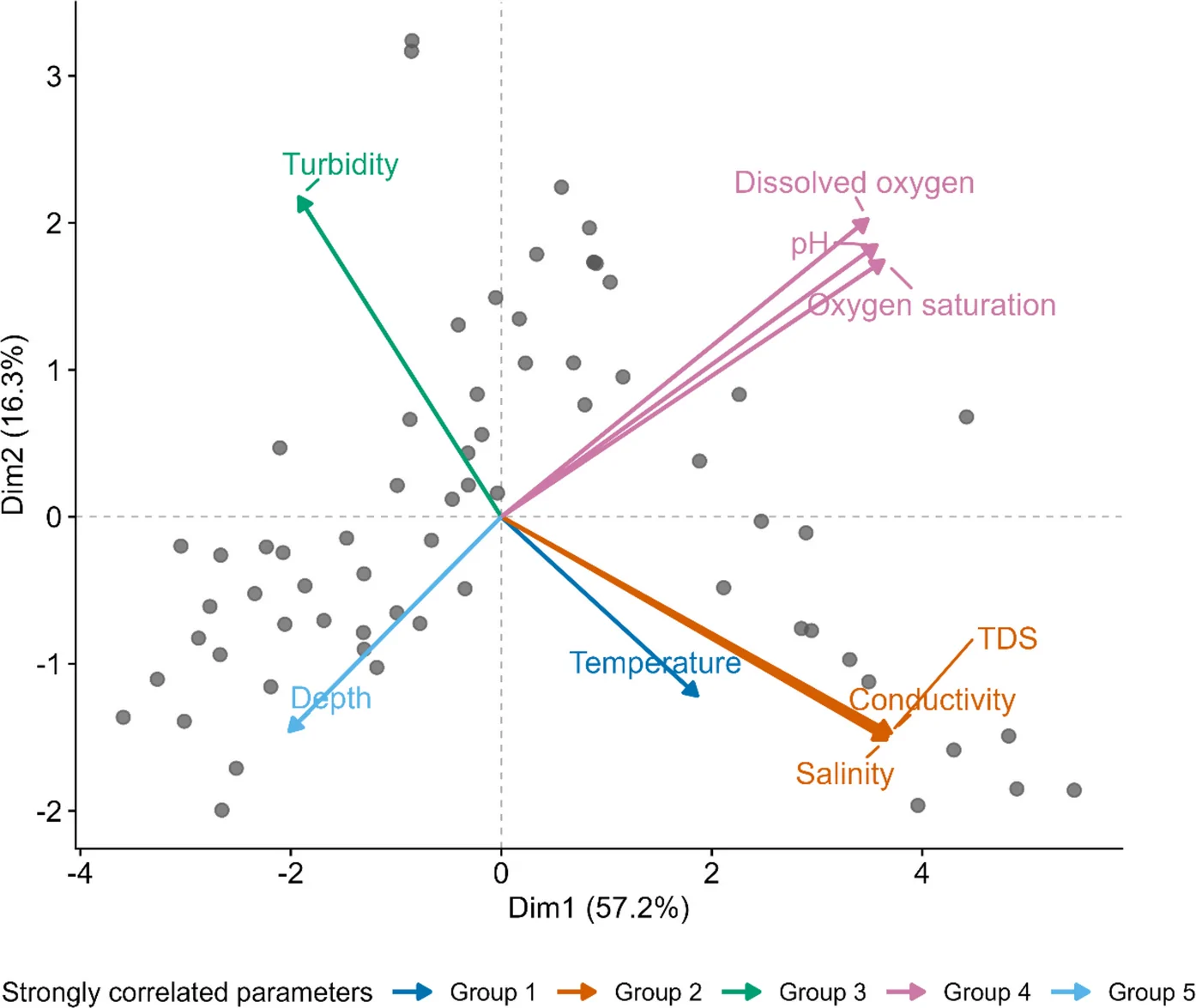
Principal component analysis (PCA) of physicochemical variables measured across the sampling stations along the Sô River–Lake Nokoué continuum

Principal component analysis (PCA) revealed a clear structuring of the physicochemical environment along two major axes (Fig. 8). The first principal component (Dim1) explained 57.2% of the total variance, whereas the second component (Dim2) explained 16.3%, together accounting for 73.5% of the total variance.

Dim1 was primarily associated with a mineralization and salinity gradient. Salinity, conductivity, and total dissolved solids (TDS) showed closely aligned vectors and strong positive associations with Dim1, indicating a high degree of covariation among these parameters. Temperature was also positively associated with Dim1, although its vector was less closely aligned with the salinity–conductivity–TDS group. In contrast, depth was oriented toward negative values of Dim1, while turbidity showed a strong negative association with Dim1.

Dim2 was mainly structured by turbidity and oxygenation-related variables. Dissolved oxygen, oxygen saturation, and pH showed closely aligned vectors toward positive Dim2 values, indicating strong positive associations among these parameters. Turbidity was also positively associated with Dim2 but was oriented toward negative Dim1 values, contrasting with the salinity–conductivity–TDS gradient. Depth was negatively associated with both axes.

This pattern indicates a pronounced fluvial–lagoon gradient in the study area. The strong contributions of salinity, electrical conductivity, and total dissolved solids (TDS) suggest that water mineralization is a major factor distinguishing the riverine and lagoonal subsystems. This finding is consistent with previous research showing that freshwater inflows from the Sô River and other tributaries contribute to regulating the physicochemical conditions of Lake Nokoué (Sintondji et al., 2022), while anthropogenic pressures also affect the lake’s water quality (Zandagba et al., 2016). Similar relationships between zooplankton community structure and physicochemical conditions have been reported in Kodjoboué Lagoon, Côte d’Ivoire, where zooplankton diversity was investigated in relation to physicochemical parameters, including conductivity, temperature, dissolved oxygen, and pH (Monney et al., 2025). Overall, the PCA highlights the main physicochemical gradients across the study area, providing an environmental context for interpreting the patterns in zooplankton and bivalve community composition examined using redundancy analysis (RDA).

### Redundancy analysis

**Fig. 9:**
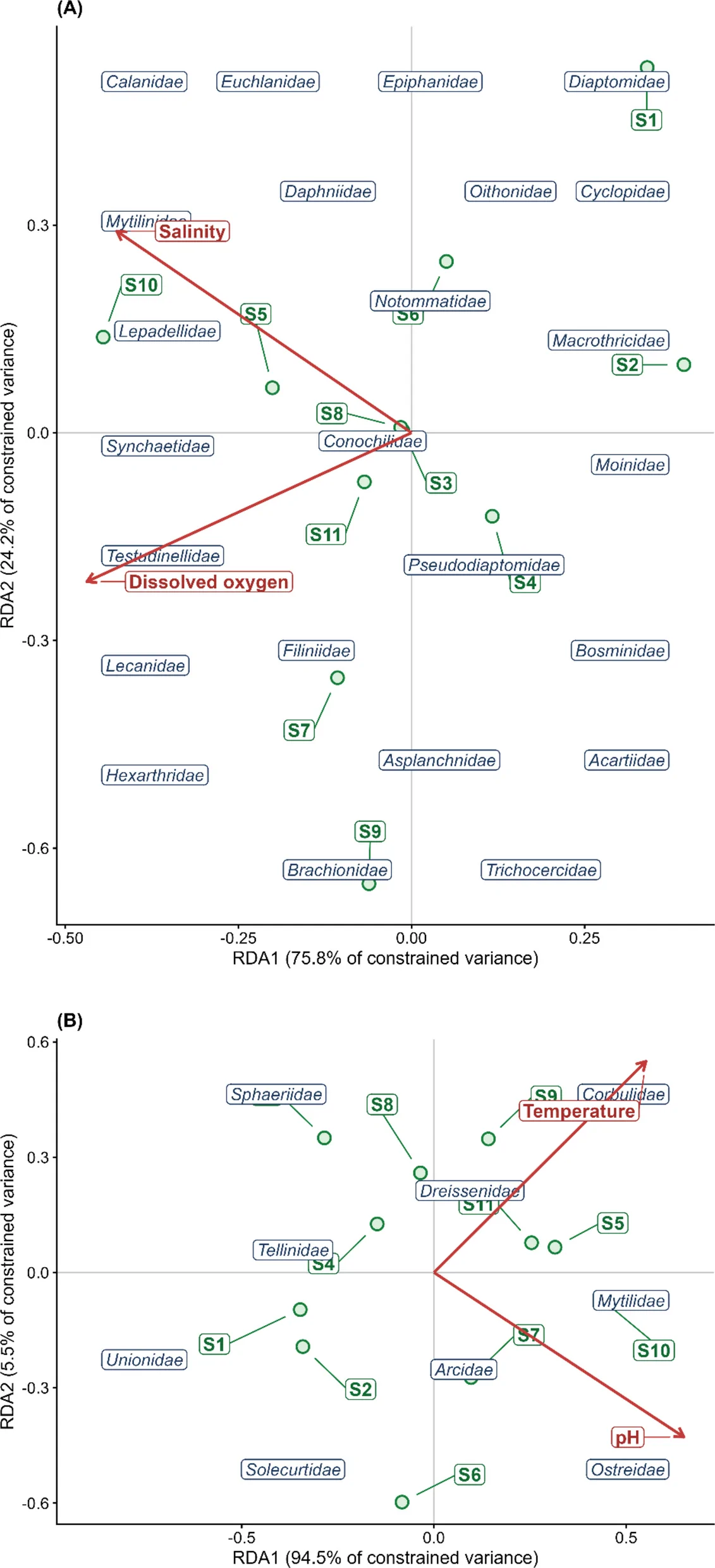
Redundancy analysis (RDA) biplots showing the relationships between selected physicochemical variables and biological community composition. **A** Zooplankton community composition in relation to salinity and dissolved oxygen. **B** Bivalve community composition in relation to temperature and pH

The RDA of zooplankton community composition retained dissolved oxygen and salinity as environmental predictors following forward selection (Fig. 9A). The selected model was statistically significant (*F* = 3.20, *P* = 0.001), explaining 9.36% of the total variation in community composition (adjusted *R*^2^ = 6.43%). Marginal permutation tests indicated significant associations with dissolved oxygen (*F* = 2.60, *P* = 0.033) and salinity (*F* = 2.12, *P* = 0.039). Both predictors had a variance inflation factor (VIF) of 1.36, indicating low multicollinearity. The first canonical axis was significant (*P* = 0.001), whereas the second was not (*P* = 0.132). RDA1 accounted for 75.8% of the constrained variance and RDA2 for 24.2%. Salinity and dissolved oxygen vectors pointed toward negative RDA1 values. Lepadellidae and Synchaetidae were positioned on the negative side of RDA1, whereas Cyclopidae, Diaptomidae, and Macrothricidae occurred on the positive side, suggesting differences in their distributions along the main ordination gradient. Despite these associations, the model explained a relatively small proportion of total community variation, suggesting that other environmental or ecological factors may also influence zooplankton distribution.

For bivalve assemblages, forward selection retained pH and temperature as predictors in the reference RDA model (Fig. 9B). The model was statistically significant (*F* = 10.10, *P* = 0.001), explaining 24.27% of the total variation in community composition (adjusted *R*^2^ = 21.87%). Marginal permutation tests indicated significant associations with pH (*F* = 10.06, *P* = 0.001) and temperature (*F* = 6.55, *P* = 0.004). Both predictors had a VIF of 1.04, indicating negligible multicollinearity. The first canonical axis was significant (*P* = 0.001), whereas the second was not (*P* = 0.430). RDA1 explained 94.5% of the constrained variance and RDA2 accounted for 5.5%. Temperature and pH vectors pointed toward positive RDA1 values but in opposite directions along RDA2. Corbulidae and Dreissenidae were located in the upper-right region of the ordination, whereas Mytilidae and Ostreidae occurred in the lower-right region, suggesting contrasting distribution patterns along the environmental gradients. The bivalve model explained a greater proportion of total community variation than the zooplankton model.

Overall, the RDA results indicate that zooplankton and bivalve community compositions were associated with different physicochemical gradients. Salinity and dissolved oxygen characterized the selected zooplankton model, whereas pH and temperature characterized the selected bivalve model. These ordination patterns indicate associations rather than causal relationships.

### Ecological differentiation between the Sô River and Lake Nokoué

The ordination analyses revealed a consistent ecological differentiation between the Sô River and Lake Nokoué, characterized by a transition from freshwater-influenced to more mineralized and saline conditions. Stations S1–S4 in the Sô River were generally associated with lower salinity and mineralization and greater influence of turbidity, whereas stations S5–S11 in Lake Nokoué were more closely associated with higher salinity and mineral content. This pattern is consistent with the functioning of river–lagoon systems as environmental transition zones, in which gradual changes in salinity and other physicochemical conditions can generate spatial turnover in aquatic communities rather than a sharp ecological boundary (Attrill & Rundle, 2002).

The observed differentiation is also consistent with the hydrological functioning of Lake Nokoué. Freshwater inflows from the Sô and other tributaries contribute to the dilution of lagoon waters during the high-water period, whereas marine intrusion through the Cotonou Channel increases salinity during periods of lower freshwater discharge (Sintondji et al., 2022). Recent modeling and field-based studies have similarly shown that river discharge and tidal exchange are major processes controlling the spatial and seasonal salinity dynamics of Lake Nokoué (Akodogbo et al., 2024; Chaigneau et al., 2023). These hydrological processes provide a plausible environmental basis for the gradient observed in the present study.

The biological differentiation observed along this gradient was particularly evident for zooplankton. Freshwater-associated families, including Cyclopidae, Moinidae, and Bosminidae, were more closely associated with the less mineralized conditions of the Sô River, whereas families such as Synchaetidae and Lecanidae were positioned toward the more mineralized conditions of the lagoon. This pattern is consistent with the findings of Chaigneau et al. (2023), who identified salinity as a major environmental factor associated with seasonal variation in zooplankton communities in Lake Nokoué. Similarly, Akodogbo et al. (2024) reported substantial changes in zooplankton abundance and composition along tidal and salinity gradients in the Cotonou Channel, demonstrating the sensitivity of planktonic communities to short-term hydrodynamic changes.

A comparable ecological differentiation was observed for bivalves. Freshwater-associated Unionidae occurred toward the less mineralized part of the ordination, whereas Corbulidae, Mytilidae, and Ostreidae were more closely associated with the mineralized and transitional lagoonal conditions. Although family-level identification prevents species-specific ecological preferences from being established, the overall pattern is consistent with studies showing that salinity, sediment characteristics, habitat structure, and hydrographic regime can strongly influence the spatial organization of benthic assemblages in coastal lagoons (Boutoumit et al., 2021). Such environmental filtering can generate distinct assemblages along freshwater–brackish gradients while maintaining substantial overlap among taxa at intermediate conditions.

The coexistence of freshwater-associated and more salinity-tolerant taxa across the study area therefore supports the interpretation of the Sô River–Lake Nokoué system as an ecological continuum rather than a discrete freshwater–lagoon boundary. The observed transition is likely shaped by the interaction of freshwater discharge, saline intrusion, hydrodynamic exchange, and local habitat conditions. Importantly, the present study demonstrates statistical associations between community composition and physicochemical gradients but does not establish causal effects of individual environmental variables. Longer-term sampling covering multiple hydrological years and incorporating sediment characteristics, nutrients, chlorophyll-a, and microbiological parameters would help disentangle the relative contributions of these processes.

### Taxonomic resolution and ecological patterns

Although family-level taxonomic resolution limits the detection of fine-scale, species-specific preferences, our results demonstrate that it can capture broad macroecological patterns along the river–lagoon continuum. In tropical socioecological systems where rapid environmental assessment is required, family-level patterns in Brachionidae and Cyclopidae may provide useful broad-scale information for preliminary environmental assessment. However, these patterns should be interpreted as ecological associations rather than species-specific responses.

### Implications for public health and climate-sensitive waterborne diseases

The environmental dynamics along the Sô River–Lake Nokoué continuum have implications for environmental health and the monitoring of climate-sensitive diseases. The occurrence of rotifers, copepods, and filter-feeding bivalves reflects the biological response of the system to environmental variability and may provide useful ecological information for monitoring. Copepods can serve as environmental hosts or reservoirs for *Vibrio cholerae* and are therefore of interest in cholera ecology (Colwell, 1996; Huq et al., 1983). The environmental conditions recorded at some stations may overlap with those previously associated with Vibrio ecology; however, the occurrence of copepods alone provides no evidence of *V. cholerae* presence or of an increased risk of cholera. However, this study did not directly assess *Vibrio cholerae* or other pathogens, so these observations should not be interpreted as evidence of pathogen occurrence or disease transmission. Rotifer abundance may also respond to changes in organic matter and dissolved nutrients, although these variables were not directly measured in the present study. Bivalves are relevant environmental indicators because filter-feeding organisms can accumulate microorganisms, suspended particles, and contaminants (Gosling, 2015; Vaughn, 2018). Their distribution across the study area therefore provides a potential biological component for environmental monitoring. The observed associations between salinity, temperature, dissolved oxygen, pH, and community structure further suggest that hydroclimatic variability may influence ecosystem functioning. Integrating biological indicators with physicochemical, microbiological, and epidemiological measurements could strengthen early-warning frameworks for climate-sensitive waterborne diseases in Benin and other tropical coastal environments.

## Study limitations

While this study can provide useful insights into the ecological landscapes of zooplankton and bivalve communities on the Sô River–Lake Nokoué continuum, there are some limitations. For one thing, the study was carried out over 8 months and may not capture the seasonal variations of environmental conditions as well as of biological communities. If the monitoring was to continue for many years, the ecology of long-term systems would be better appreciated. Secondly, taxonomic analysis was done at the family level and species-specific responses to changes in the environment might have been overlooked. Thirdly, although the physicochemical conditions were an important factor for the formation of the communities, nutrients, sediment, chlorophyll-a, hydrological connections, and microbial communities were not assessed. Finally, although these ecological findings may be relevant to future environmental health research, this study did not directly assess the presence of pathogenic microorganisms, such as *Vibrio cholerae*. Therefore, the observed community patterns should not be interpreted as evidence of pathogen occurrence, human exposure, or disease risk. On the other hand, more comprehensive ecological, microbiological, molecular, and epidemiological data over longer monitoring periods will help us to understand ecosystem functioning and climate-sensitive waterborne disease risk.

## Conclusion

This study characterized zooplankton and bivalve communities along the Sô River–Lake Nokoué continuum and documented associations between community composition and physicochemical gradients. Zooplankton assemblages were dominated by Eurotatoria, whereas bivalves exhibited lower family-level richness but occurred across multiple stations. Multivariate analyses distinguished freshwater-influenced river stations from more saline lagoon stations, with salinity and related physicochemical variables associated with community variation. These findings support the potential value of zooplankton and bivalve monitoring for characterizing environmental change in tropical river–lagoon systems. However, because pathogens, human exposures, and disease outcomes were not investigated, the results do not establish a relationship between the observed communities and waterborne disease risk. Further studies integrating ecological, microbiological, and epidemiological measurements are needed before these communities can be evaluated as indicators within disease early-warning systems.

## Data Availability

The datasets generated and analyzed during the current study are not publicly available because they form part of ongoing research activities. However, the data supporting the findings of this study are available from the corresponding author upon reasonable request. Requests for access will be considered in accordance with institutional policies and ethical requirements.

## References

[CR1] Adandédjan, D. (2012). *Diversity and determinants of benthic macroinvertebrate communities in two lagoons of southern Benin: Porto-Novo Lagoon and the Coastal Lagoon (doctoral dissertation)*. University of Abomey-Calavi.

[CR2] Adandédjan, D., Makponse, E., Hinvi, L. C., & Laleye, P. (2017). Preliminary data on the diversity of zooplankton in Lake Nokoué (Southern Benin). *Journal of Applied Biosciences,**115*(1), 11476–11486. 10.4314/jab.v115i1.7

[CR3] Aggio, C. E. G., Oliveira, F. R., Progênio, M., Bello, J. R., Lansac-Tôha, F. M., & Velho, L. F. M. (2022). The zooplankton of tropical streams: Is it determinism or stochasticity that drives the spatial and temporal patterns in community structure? *Community Ecology,**23*(2), 219–229. 10.1007/s42974-022-00099-2

[CR4] Akodogbo, H. H., Dossou-Sognon, F. U., Ouinsou, F. T., Avocegan, T. T., Kouglo, J. P., Okpeitcha, O. V., Assogba, A., Sohou, Z., Morel, Y., & Chaigneau, A. (2024). Tidal impacts on zooplankton dynamics in a major ocean–lagoon channel: Insights from a 25-hour intensive survey in the Cotonou Channel, Benin. *Journal of Marine Science and Engineering,**12*(9), Article 1519. 10.3390/jmse12091519

[CR5] Allan, J. D., & Castillo, M. M. (2007). *Stream ecology*. Springer Netherlands. 10.1007/978-1-4020-5583-6

[CR6] Attrill, M. J., & Rundle, S. D. (2002). Ecotone or ecocline: Ecological boundaries in estuaries. *Estuarine, Coastal and Shelf Science,**55*(6), 929–936. 10.1006/ecss.2002.1036

[CR7] Bekele, B. K., Uwishema, O., Bisetegn, L. D., Moubarak, A., Charline, M., Sibomana, P., & Onyeaka, C. V. P. (2025). Cholera in Africa: A climate change crisis. *Journal of Epidemiology and Global Health,**15*(1), Article 68. 10.1007/s44197-025-00386-x PMC12043531 40304931

[CR8] Bhandarkar, S., & Bansod, S. (2025). The role of Rotifera as sentinel organism of trophic structure on freshwater ecosystems. *International Journal of Scientific Research in Biological Sciences,**12*(2), 41–52. 10.26438/ijsrbs.v12i2.677

[CR9] Bogan, A. E. (2008). Global diversity of freshwater mussels (Mollusca, Bivalvia) in freshwater. *Hydrobiologia,**595*(1), 139–147. 10.1007/s10750-007-9011-7

[CR10] Boutoumit, S., Bououarour, O., El Kamcha, R., Pouzet, P., Zourarah, B., Benhoussa, A., Maanan, M., & Bazairi, H. (2021). Spatial patterns of macrozoobenthos assemblages in a sentinel coastal lagoon: Biodiversity and environmental drivers. *Journal of Marine Science and Engineering,**9*(5), Article 461. 10.3390/jmse9050461

[CR11] Cabral, C. R., Diniz, L. P., Da Silva, A. J., Fonseca, G., Carneiro, L. S., De Melo Júnior, M., & Caliman, A. (2020). Zooplankton species distribution, richness and composition across tropical shallow lakes: A large scale assessment by biome, lake origin, and lake habitat. *Annales de Limnologie - International Journal of Limnology,**56*, Article 25. 10.1051/limn/2020023

[CR12] Capo-Chichi, H. B., Adandedjan, D., & Laleye, P. A. (2022). Updating benthic macroinvertebrates database of the Nokoue Lake in south eastern Benin. *International Journal of Science and Research (IJSR), 11*(10), 44–56. 10.21275/SR22929205839

[CR13] Chaigneau, A., Ouinsou, F. T., Akodogbo, H. H., Dobigny, G., Avocegan, T. T., Dossou-Sognon, F. U., Okpeitcha, V. O., Djihouessi, M. B., & Azémar, F. (2023). Physicochemical drivers of zooplankton seasonal variability in a West African lagoon (Nokoué Lagoon, Benin). *Journal of Marine Science and Engineering,**11*(3), Article 556. 10.3390/jmse11030556

[CR14] Colwell, R. R. (1996). Global climate and infectious disease: The cholera paradigm. *Science,**274*(5295), 2025–2031. 10.1126/science.274.5295.2025 8953025

[CR15] Dajoz, R. (2000). *Précis d’écologie [Principles of ecology]* (7th ed.). Dunod.

[CR16] Davies-Vollum, K. S., Koomson, D., & Raha, D. (2024). Coastal lagoons of West Africa: A scoping study of environmental status and management challenges. *Anthropocene Coasts,**7*(1), 7. 10.1007/s44218-024-00039-9

[CR17] de Magny, G. C., Mozumder, P. K., Grim, C. J., Hasan, N. A., Naser, M. N., Alam, M., Sack, R. B., Huq, A., & Colwell, R. R. (2011). Role of zooplankton diversity in *Vibrio cholerae* population dynamics and in the incidence of cholera in the Bangladesh Sundarbans. *Applied and Environmental Microbiology,**77*(17), 6125–6132. 10.1128/AEM.01472-10 PMC3165371 21764957

[CR18] Dehingia, R. K., Pankaj, P. P., Kakati, L. N., Gogoi, J., & Chakraborty, S. (2026). Cladocerans and copepods as bioindicators of aquatic ecosystem health: Ecological roles, anthropogenic stressors and limitations. *Ecological Indicators,**184*, Article 114712. 10.1016/j.ecolind.2026.114712

[CR19] Djihouessi, M. B., & Aina, M. P. (2018). A review of hydrodynamics and water quality of Lake Nokoué: Current state of knowledge and prospects for further research. *Regional Studies in Marine Science,**18*, 57–67. 10.1016/j.rsma.2018.01.002

[CR20] Dumont, H. J. (1994). The distribution and ecology of the fresh- and brackish-water medusae of the world. *Hydrobiologia,**272*(1–3), 1–12. 10.1007/BF00006508

[CR21] Durand, J.-R., & Lévêque, C. (Éds.). (1981). Flore et faune aquatiques de l’Afrique sahélo-soudanienne (Tome 2 : Insectes et vertébrés). Office de la recherche scientifique et technique outre-mer (ORSTOM).Erken, M., Lutz, C., & McDougald, D. (2015). Interactions of Vibrio spp. with zooplankton. *Microbiology Spectrum, 3*(3). 10.1128/microbiolspec.VE-0003-2014 26185068

[CR22] Erken, M., Lutz, C., & McDougald, D. (2015). Interactions of *Vibrio* spp. with zooplankton. *Microbiology Spectrum*. 10.1128/microbiolspec.VE-0003-2014 26185068

[CR23] Gavrilko, D., Zhikharev, V., Zolotareva, T., Kudrin, I., Yakimov, B., & Erlashova, A. (2024). Biodiversity of zooplankton (Rotifera, Cladocera and Copepoda) in the tributaries of Cheboksary Reservoir (Middle Volga, Russia). *Biodiversity Data Journal,**12*, Article e116330. 10.3897/BDJ.12.e116330 PMC10880022 38384788

[CR24] Ge, J., Chen, J., Zi, F., Song, T., Hu, L., He, Z., Wu, L., Ding, Y., & Li, H. (2025). Seasonal variations in macrobenthos communities and their relationship with environmental factors in the Alpine Yuqu River. *Biology,**14*(2), 120. 10.3390/biology14020120 PMC11852101 40001888

[CR25] Gnohossou, P. M. (2006). *La faune benthique d’une lagune ouest africaine (le Lac Nokoué au Bénin), diversité, abondance, variations temporelles et spatiales, place dans la chaîne trophique* (p. 169). Thèse de Doctorat. Institut National Polytechnique de Toulouse. Formation doctorale: SEVAB.

[CR26] Gnohossou, P., Laleye, P., Atachi, P., & Moreau, J. (2009). The role of macroinvertebrates in the diets of the dominant fish species in Lake Nokoué, Benin. *African Journal of Aquatic Science,**34*(2), 189–194. 10.2989/AJAS.2009.34.2.10.897

[CR27] Gosling, E. (2015). *Marine bivalve molluscs* (1st edn). Wiley. 10.1002/9781119045212

[CR28] Huq, A., Small, E. B., West, P. A., Huq, M. I., Rahman, R., & Colwell, R. R. (1983). Ecological relationships between *Vibrio cholerae* and planktonic crustacean copepods. *Applied and Environmental Microbiology,**45*(1), 275–283. 10.1128/aem.45.1.275-283.1983 PMC242265 6337551

[CR29] Jeppesen, E., Nõges, P., Davidson, T. A., Haberman, J., Nõges, T., Blank, K., Lauridsen, T. L., Søndergaard, M., Sayer, C., Laugaste, R., Johansson, L. S., Bjerring, R., & Amsinck, S. L. (2011). Zooplankton as indicators in lakes: A scientific-based plea for including zooplankton in the ecological quality assessment of lakes according to the European Water Framework Directive (WFD). *Hydrobiologia,**676*(1), 279–297. 10.1007/s10750-011-0831-0

[CR30] Lipp, E. K., Huq, A., & Colwell, R. R. (2002). Effects of global climate on infectious disease: The cholera model. *Clinical Microbiology Reviews,**15*(4), 757–770. 10.1128/CMR.15.4.757-770.2002 PMC126864 12364378

[CR31] Liu, P., Wang, T., Li, H., Zhang, X., Wang, L., Jeppesen, E., & Han, B.-P. (2023). Functional diversity and redundancy of rotifer communities affected synergistically by top-down and bottom-up effects in tropical urban reservoirs. *Ecological Indicators,**155*, Article 111061. 10.1016/j.ecolind.2023.111061

[CR32] Lopes-Lima, M., Sousa, R., Geist, J., Aldridge, D. C., Araujo, R., Bergengren, J., Bespalaya, Y., Bódis, E., Burlakova, L., Van Damme, D., Douda, K., Froufe, E., Georgiev, D., Gumpinger, C., Karatayev, A., Kebapçi, Ü., Killeen, I., Lajtner, J., Larsen, B. M., … Zogaris, S. (2017). Conservation status of freshwater mussels in Europe: State of the art and future challenges: Conservation of European freshwater mussels. *Biological Reviews,**92*(1), 572–607. 10.1111/brv.12244 26727244

[CR33] Mohammed, Y. M., Arimoro, F. O., Ayanwale, A. V., Adama, B. S., Keke, U. N., Auta, Y. I., & Umar, B. I. (2020). Seasonal changes in the abundance of benthic macroinvertebrates & physico-chemical condition of Moussa stream Bida, Nigeria. *Tropical Freshwater Biology,**29*(1), 57–70. 10.4314/tfb.v29i1.4

[CR34] Moisan, J. (2010). *Guide d’identification des principaux macroinvertébrés benthiques d’eau douce du Québec: Surveillance volontaire des cours d’eau peu profonds* (p. 82). Direction du suivi de l’état de l’environnement, ministère du Développement durable, de l’Environnement et des Parcs.

[CR35] Monney, I. A., N’Dri, O. R., Dago, G.-M., Konan, Y. A., & Koné, T. (2025). Influence of physico-chemical parameters on the diversity of zooplankton in Kodjoboué lagoon (Bonoua, south-east of Côte d’Ivoire). *World Journal of Advanced Research and Reviews,**28*(2), 1388–1397. 10.30574/wjarr.2025.28.2.3817

[CR36] Monney, I. A., Ouattara, I. N., Etilé, R. N., Aka, M. N., Bamba, M., & Koné, T. (2016). Distribution of zooplankton in relation to the environmental characteristics of four coastal rivers in southeastern Côte d’Ivoire (West Africa). *Journal of Applied Biosciences,**98*(1), 9344–9358. 10.4314/jab.v98i1.10

[CR37] Perera, I. U., Fujiyoshi, S., Nishiuchi, Y., Nakai, T., & Maruyama, F. (2022). Zooplankton act as cruise ships promoting the survival and pathogenicity of pathogenic bacteria. *Microbiology and Immunology,**66*(12), 564–578. 10.1111/1348-0421.13029 PMC10091822 36128640

[CR38] Phan, N.-T., Duong, Q. H., Tran-Nguyen, Q. A., & Trinh-Dang, M. (2021). The species diversity of tropical freshwater rotifers (Rotifera: Monogononta) in relation to environmental factors. *Water,**13*(9), Article 1156. 10.3390/w13091156

[CR39] Ramos, E. A., De Morais-Junior, C. S., Rodrigues-Filho, C. A. S., Sánchez-Botero, J. I., Melo Júnior, M., & Novaes, J. L. C. (2022). Influence of spatial and environmental factors on the structure of a zooplankton metacommunity in an intermittent river. *Aquatic Ecology,**56*(1), 239–249. 10.1007/s10452-021-09912-y

[CR40] Segers, H. (2008). Global diversity of rotifers (Rotifera) in freshwater. *Hydrobiologia,**595*(1), 49–59. 10.1007/s10750-007-9003-7

[CR41] Sintondji, S. W., Sohou, Z., Baetens, K., Lacroix, G., & Fiogbé, E. D. (2022). Characterization of a West African coastal lagoon system: Case of Lake Nokoué with its inlet (Cotonou, South Benin). *Ecologies,**3*(4), 467–479. 10.3390/ecologies3040033

[CR42] Sousa, R., Novais, A., Costa, R., & Strayer, D. L. (2014). Invasive bivalves in fresh waters: Impacts from individuals to ecosystems and possible control strategies. *Hydrobiologia,**735*(1), 233–251. 10.1007/s10750-012-1409-1

[CR43] Stamou, G., Katsiapi, M., Demertzioglou, M., Voutsa, D., Kozari, A., Pantelaki, I., Moustaka-Gouni, M., & Michaloudi, E. (2023). Pelagial zooplankton community in a newly established reservoir during and after the impoundment of a hydropower dam. *Diversity,**15*(2), 257. 10.3390/d15020257

[CR44] Strayer, D. L. (2008). *Freshwater mussel ecology: A multifactor approach to distribution and abundance*. University of California Press.

[CR45] Tachet, H., Richoux, P., Bournaud, M., & Usseglio-Polatera, P. (2006). *Invertébrés d’eau douce: Systematique, biologie, ecologie* (p. 588). CNRS 2eme Editions.

[CR46] Umi, W. A. D., Yusoff, F. M., Balia Yusof, Z. N., Ramli, N. M., Sinev, A. Y., & Toda, T. (2024). Composition, distribution, and biodiversity of zooplanktons in tropical lentic ecosystems with different environmental conditions. *Arthropoda,**2*(1), 33–54. 10.3390/arthropoda2010003

[CR47] Vaughn, C. C. (2018). Ecosystem services provided by freshwater mussels. *Hydrobiologia,**810*(1), 15–27. 10.1007/s10750-017-3139-x

[CR48] Vezzulli, L., Grande, C., Reid, P. C., Hélaouët, P., Edwards, M., Höfle, M. G., Brettar, I., Colwell, R. R., & Pruzzo, C. (2016). Climate influence on *Vibrio* and associated human diseases during the past half-century in the coastal North Atlantic. *Proceedings of the National Academy of Sciences of the United States of America*. 10.1073/pnas.1609157113 PMC5003230 27503882

[CR49] Wagaw, S., Mengistou, S., & Getahun, A. (2023). Zooplankton community structure in relation to environmental variables in a tropical endorheic soda lake Shala, Ethiopia. *Journal of Freshwater Ecology,**38*(1), Article 2188882. 10.1080/02705060.2023.2188882

[CR50] World Health Organization. (2023). *Cholera – global situation*. https://www.who.int/emergencies/disease-outbreak-news/item/2023-DON437. Accessed 7 Nov 2026.

[CR51] Zandagba, J., Adandedji, F. M., Mama, D., Chabi, A., & Afouda, A. (2016). Assessment of the physicochemical pollution of a water body in a perspective of integrated water resource management: Case study of Lake Nokoué. *Journal of Environmental Protection,**7*(5), 656–669. 10.4236/jep.2016.75059

